# Albuminuria Levels and Geriatric Outcomes in Predialysis: Chronic Kidney Disease: Falls, Fear of Falling, and Frailty in a Cross-Sectional Study

**DOI:** 10.3390/jcm15124772

**Published:** 2026-06-19

**Authors:** Vedat Gençer, Yavuz Sultan Selim Akgül, Burcu Eren Cengiz, İsmail Altıntop

**Affiliations:** 1 Division of Nephrology, Department of Internal Medicine, Kayseri City Training and Research Hospital, University of Health Sciences, 38080 Kayseri, Türkiye; 2 Division of Geriatrics, Department of Internal Medicine, Kayseri City Training and Research Hospital, University of Health Sciences, 38080 Kayseri, Türkiye; yavuzssakgul@gmail.com (Y.S.S.A.); burcuerencengiz@gmail.com (B.E.C.); 3 Department of Emergency Medicine, Kayseri City Training and Research Hospital, University of Health Sciences, 38080 Kayseri, Türkiye; ismail.altintop@sbu.edu.tr

**Keywords:** albuminuria, chronic kidney disease, falls, frailty, geriatric outcomes, predialysis, inflammatory indices

## Abstract

**Background:** Chronic kidney disease (CKD) accelerates biological aging and amplifies the risk of adverse geriatric outcomes. Albuminuria reflects systemic endothelial dysfunction beyond renal damage, yet its specific relationship with falls, fear of falling, and frailty in predialysis CKD patients remains underexplored. **Objectives:** We aimed to evaluate the association between albuminuria levels (urinary albumin-to-creatinine ratio, ACR) with falls, fear of falling (Falls Efficacy Scale, FES), and frailty (FRAIL scale and Clinical Frailty Scale, CFS) in older adults with CKD. **Methods:** This cross-sectional study analyzed 295 patients aged ≥60 years attending nephrology and geriatrics clinics at Kayseri City Hospital, Turkey (April–June 2025). ACR was categorized per KDIGO (A1: <30, A2: 30–300, A3: ≥300 mg/g). Inflammatory indices (NLR, SII, CAR) were calculated. Hierarchical multivariable logistic regression and ROC analyses were performed. **Results:** Fall prevalence showed a clear dose-response across ACR categories: 31.2% (A1), 72.0% (A2), and 93.2% (A3) (*p* < 0.001). In the fully adjusted model, each unit increase in log-ACR was associated with a 3.84-fold increase in fall odds (OR 3.84, 95% CI 2.74–6.65). Although bivariate ACR-frailty associations were non-significant, fully adjusted models uncovered independent associations across both instruments and thresholds: FRAIL ≥ 3 (OR 1.41, 95% CI 1.05–2.03), FRAIL ≥ 2 (OR 1.49, 95% CI 1.08–2.21), CFS ≥ 5 (OR 1.87, 95% CI 1.38–2.83), and CFS ≥ 4 (OR 1.37, 95% CI 1.02–1.93). ACR showed good discriminative ability for falls (AUC 0.773, optimal cut-off 21.70 mg/g) but poor discrimination for frailty (AUC 0.50–0.54). The ACR–fall association was stronger in patients with GFR < 60 (OR 4.48) than GFR ≥ 60 (OR 2.18). **Conclusions:** Albuminuria is a strong, independent, and graded predictor of falls in older CKD patients, with a nearly 4-fold increase in risk per log-unit ACR increase after full adjustment. ACR measurement, already routine in CKD monitoring, could help identify older patients at increased fall risk and guide targeted geriatric assessment. However, ACR showed poor standalone discriminative ability for frailty across all definitions (AUC 0.50–0.54), establishing that it cannot serve as a frailty screening tool in isolation.

## 1. Introduction

Chronic kidney disease (CKD) represents a growing global health burden, affecting approximately 10–15% of the adult population worldwide and disproportionately impacting older individuals [1,2]. In the predialysis stages, CKD is not merely a disorder of declining filtration capacity; rather, it is a systemic condition associated with accelerated biological ageing, driven in part by cellular senescence, telomere attrition, oxidative stress, and persistent uraemic inflammation, thereby increasing vulnerability to adverse geriatric outcomes, including frailty and falls [3,4,5,6]. Falls constitute the leading cause of injury-related morbidity and mortality in older adults, and patients with CKD experience fall rates substantially higher than age-matched individuals with preserved kidney function [5,6,7]. Frailty, characterized by diminished physiological reserve and heightened vulnerability to stressors, is recognized as a strong independent predictor of hospitalization, disability, and mortality in CKD populations [5,8,9].

Albuminuria, quantified as the urinary albumin-to-creatinine ratio (ACR), has traditionally been utilized as a marker of glomerular barrier integrity and CKD progression [10]. However, growing evidence points to albuminuria extending beyond its role as a renal biomarker; it reflects systemic endothelial dysfunction, chronic low-grade inflammation, and microvascular damage that may contribute to extrarenal pathology [11,12]. The Kidney Disease Improving Global Outcomes (KDIGO) classification stratifies albuminuria into three categories: A1 (normal to mildly increased, ACR < 30 mg/g), A2 (moderately increased, ACR 30–300 mg/g, formerly termed microalbuminuria), and A3 (severely increased, ACR ≥ 300 mg/g, formerly termed macroalbuminuria), each carrying incrementally higher risks for adverse cardiovascular and renal outcomes [13]. Whether this stepwise increase in risk extends to geriatric outcomes in predialysis CKD patients is clinically important but largely unanswered.

An albuminuria–falls–frailty axis is supported by biology. Albuminuria correlates with systemic inflammation, as reflected by elevated C-reactive protein (CRP), neutrophil-to-lymphocyte ratio (NLR), and systemic immune–inflammation index (SII) [14,15]. These inflammatory mediators promote sarcopenia, neuromuscular dysfunction, and cognitive decline, all of which are mechanistic contributors to both falls and frailty [16,17]. Albuminuria is also associated with reduced serum albumin, vitamin D deficiency, and metabolic acidosis, each independently linked to musculoskeletal impairment and functional decline [16,17,18].

Several studies have explored the relationship between kidney function markers and geriatric outcomes. Bowling et al. found that reduced eGFR and elevated albuminuria were independently associated with serious fall injuries in over 16,000 community-dwelling older adults [6]. Mielke et al. reported that even mildly elevated albuminuria predicted frailty worsening and death in very old adults [19]. Chang et al. identified low-grade albuminuria as an independent predictor of pre-frailty/frailty among community-dwelling adults in Taiwan [20]. Bongetti et al. found that albuminuria, but not eGFR, was associated with incident frailty among initially healthy older adults in the ASPREE trial [21]. A recent individual patient-level meta-analysis by Heerspink et al. involving nearly 149,000 participants compared ACR and urinary protein-to-creatinine ratio (PCR) for predicting kidney failure, establishing ACR as the superior biomarker [22].

Despite these advances, critical gaps persist. Most existing studies have examined eGFR as the primary exposure, with albuminuria as a secondary variable [6]. Few studies have simultaneously used dual frailty assessment tools to cross-validate findings [4,8]. The potential mediating role of novel inflammatory indices (NLR, SII, CAR) in the albuminuria–frailty pathway has not been adequately explored [15,23]. Finally, no study from Turkey has addressed the albuminuria–falls–frailty nexus in predialysis CKD patients using validated geriatric assessment tools.

We designed this cross-sectional study to: (1) determine the prevalence of falls, fear of falling, and frailty stratified by KDIGO albuminuria categories; (2) evaluate whether ACR is independently associated with these outcomes after adjusting for demographic, clinical, and laboratory confounders; (3) assess the discriminative performance of ACR using ROC analysis; and (4) explore the contribution of inflammatory indices to the albuminuria–geriatric outcome relationship.

## 2. Materials and Methods

### 2.1. Study Design and Setting

This single-center, observational, analytical, cross-sectional, exploratory study was conducted at the Nephrology and Geriatrics Outpatient Clinics of Kayseri City Training and Research Hospital, a tertiary care facility affiliated with the University of Health Sciences, Kayseri, Turkey. Data collection took place between 3 April and 30 June 2025. The study was approved by the Institutional Non-Interventional Clinical Research Ethics Committee and conducted in accordance with the Declaration of Helsinki. Written informed consent was obtained from all participants. This manuscript adheres to the STROBE guidelines for cross-sectional studies [24].

### 2.2. Study Population

**Figure 1:**
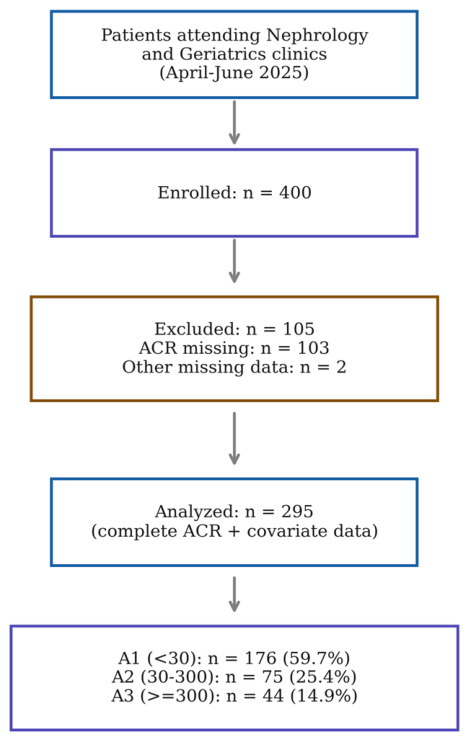
Study flow diagram. Of 400 enrolled patients, 295 with complete ACR and covariate data were included in the final analysis.

We enrolled 400 consecutive patients aged 60 years and older presenting to the nephrology and geriatrics outpatient clinics during the study period. Inclusion criteria were: (a) age ≥ 60 years; (b) follow-up at the nephrology and/or geriatrics clinic within the preceding six months. Exclusion criteria were: (a) acute kidney injury; (b) advanced dementia precluding reliable questionnaire responses; and (c) major trauma, fracture, or hospitalization within the preceding three months. Of the 400 enrolled patients, 105 were excluded from the final analysis due to missing ACR data (*n* = 103) or incomplete covariate information (*n* = 2), yielding an analytic cohort of 295 participants (Figure 1). A comparison of baseline characteristics between included and excluded participants is provided in Table S1. In routine clinical practice, spot urine samples may not be obtained from elderly patients due to compliance difficulties, urinary incontinence, or because ACR testing was not specifically requested for patients with preserved kidney function who were primarily followed in the geriatrics clinic. Nephrology clinic patients had clinically documented CKD according to KDIGO criteria, and acute kidney injury was excluded. For patients recruited from the geriatrics clinic, reduced eGFR, when present, was verified from prior clinical records; however, among geriatrics clinic participants with preserved eGFR, albuminuria-based CKD classification relied on the available spot ACR measurement because repeat ACR confirmation was not systematically available.

### 2.3. Exposure Variable

Albuminuria was measured using the spot urine albumin-to-creatinine ratio (ACR, mg/g) and categorized per KDIGO 2012 guidelines: A1 (normal to mildly increased, <30 mg/g), A2 (moderately increased, 30–300 mg/g), and A3 (severely increased, ≥300 mg/g) [13]. ACR was also analyzed as a continuous variable after log10 transformation due to right-skewed distribution.

### 2.4. Outcome Variables

**Falls:** The number of falls within the preceding 12 months was recorded via patient and caregiver report. Falls were analyzed as a binary variable (faller ≥ 1 vs. non-faller) and as a count variable.

**Fear of falling:** The Falls Efficacy Scale (FES) was administered (range 10–100, higher = greater fear). A cut-off of ≥70 defined clinically significant fear of falling [25].

**Frailty (FRAIL scale):** Scores 0–5; categorized as robust (0), pre-frail (1–2), or frail (≥3) [26].

**Frailty (Clinical Frailty Scale, CFS):** Scores 1–9; categorized as fit (1–3), pre-frail (4), or frail (5–9) [27].

### 2.5. Covariates and Inflammatory Indices

Demographic variables included age, sex, and education level. Clinical variables included comorbidities (diabetes mellitus [DM], hypertension [HT], coronary artery disease, and others), total comorbidity count, and polypharmacy (≥4 medications) [28]. Laboratory parameters included serum creatinine, estimated GFR (CKD-EPI equation), serum albumin, CRP, hemoglobin, and complete blood count. Inflammatory indices were calculated: NLR (neutrophil/lymphocyte), SII (platelet × neutrophil/lymphocyte), and CAR (CRP/albumin) [14,15,23].

### 2.6. Statistical Analysis

All analyses were performed using IBM SPSS Statistics version version (26.0; IBM Corp., Armonk, NY, USA) with verification in Python (version 3.11; Python Software Foundation, https://www.python.org). Continuous variables were expressed as median (IQR) and compared using the Kruskal–Wallis test with Dunn post-hoc correction. Categorical variables were compared using chi-square test with Cramer V effect size. Spearman correlations were calculated between ACR and all outcomes. Hierarchical logistic regression was performed: Model 0 (crude), Model 1 (adjusted for age and sex), and Model 2 (fully adjusted for age, sex, GFR, DM, HT, polypharmacy, comorbidity count, and education). Bootstrap confidence intervals (1000 resamples) were calculated. ROC curves with AUC (95% CI) and Youden-index optimal cut-offs were generated. Subgroup analyses were stratified by GFR (<60 vs. ≥60), sex, and DM status. Sensitivity analysis was restricted to patients with GFR < 60 mL/min. Significance was set at *p* < 0.05 (two-tailed). Benjamini–Hochberg false discovery rate (FDR) correction was applied across all primary regression analyses to control for multiple comparisons. Post-hoc power analysis confirmed > 99.9% power for the primary comparison (A1 31.2% vs. A3 93.2%, *n* = 220) and for A1 vs. A2 (31.2% vs. 72.0%, *n* = 251) at alpha = 0.05.

## 3. Results

### 3.1. Baseline Characteristics

Of 400 enrolled patients, 295 had complete ACR and covariate data and comprised the analytic cohort (Figure 1). The median age was 73 years (IQR 68–80), 65.4% were female, and 45.4% were illiterate. The median GFR was 55 mL/min/1.73 m^2^ (IQR 39–77), with 57.6% having GFR < 60. DM was present in 54.2%, HT in 81.7%, and polypharmacy in 51.2%. By KDIGO albuminuria category, 176 (59.7%) were A1, 75 (25.4%) were A2, and 44 (14.9%) were A3. Excluded participants had significantly higher GFR (median 80 vs. 55, *p* < 0.001), lower fall prevalence (32.3% vs. 50.8%, *p* = 0.002), and lower DM prevalence (40.0% vs. 54.2%, *p* = 0.017), confirming non-random missingness concentrated among lower-risk patients (Table S1).

Participants with higher ACR categories had significantly lower GFR (A1: 66, A2: 43, A3: 32 mL/min; *p* < 0.001), lower serum albumin (*p* < 0.001), higher CRP (*p* < 0.001), higher NLR (*p* = 0.001), higher SII (*p* < 0.001), and lower hemoglobin (*p* < 0.001). Age was slightly but significantly lower in A3 (median 70 vs. 73–75 years; *p* = 0.024). Sex distribution and diabetes prevalence did not differ significantly across groups.

### 3.2. Prevalence of Falls and Frailty

Half the cohort (150/295, 50.8%) had fallen at least once in the preceding year. Clinically significant fear of falling (FES ≥ 70) affected 23.4%. By the FRAIL scale, 42.4% qualified as frail (≥3) and 31.8% by the CFS (≥5). An additional 46.9% and 31.5% were classified as pre-frail by the respective instruments.

**Figure 2:**
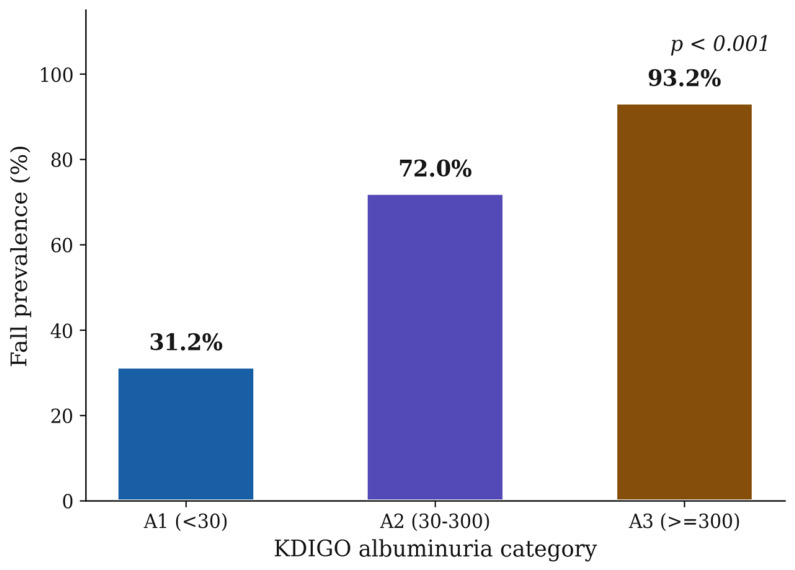
Fall prevalence (%) by KDIGO albuminuria category. A dose–response relationship is evident: A1 31.2%, A2 72.0%, A3 93.2% (chi-square = 72.02, *p* < 0.001). *n* = 295.

**Figure 3:**
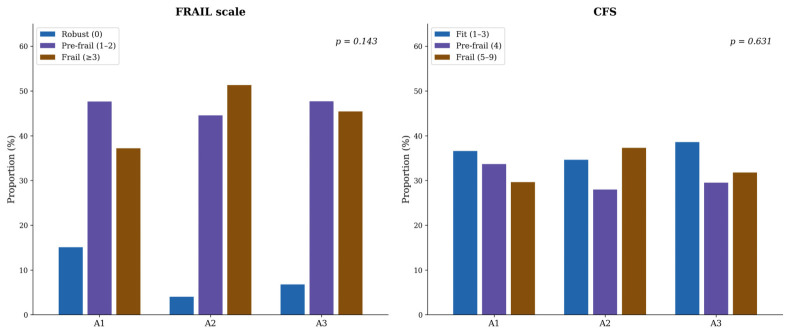
Frailty distribution by ACR category using the FRAIL scale ((**left**), *p* = 0.143) and Clinical Frailty Scale ((**right**), *p* = 0.631). *n* = 295.

Fall prevalence demonstrated a clear dose–response gradient across ACR categories: 31.2% in A1, 72.0% in A2, and 93.2% in A3 (chi-square = 72.02, *p* < 0.001; Figure 2). FES scores followed a similar gradient (A1: 30, A2: 55, A3: 79; *p* < 0.001). FRAIL and CFS scores trended toward higher frailty of increasing frailty across ACR categories, but neither reached statistical significance without adjustment (FRAIL: *p* = 0.143; CFS: *p* = 0.631; Figure 3).

### 3.3. Correlation Analysis

**Figure 4:**
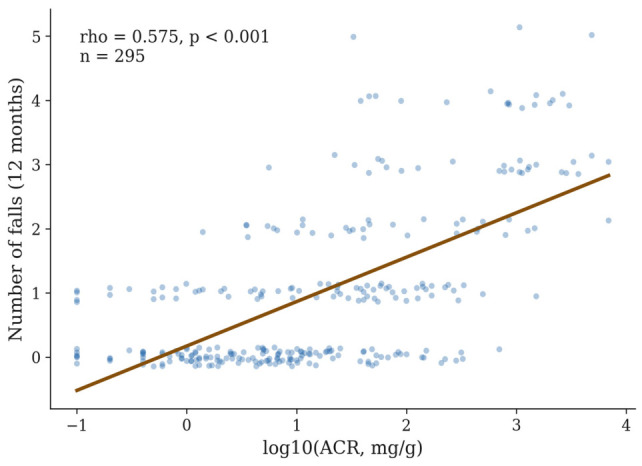
Scatter plot of log10(ACR) versus number of falls with regression line. Spearman rho = 0.575, *p* < 0.001. *n* = 295.

**Figure 5:**
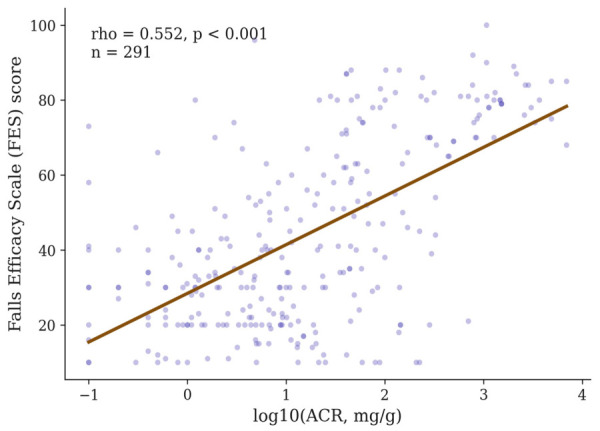
Scatter plot of log10(ACR) versus FES score with regression line. Spearman rho = 0.552, *p* < 0.001. *n* = 291.

**Figure 6:**
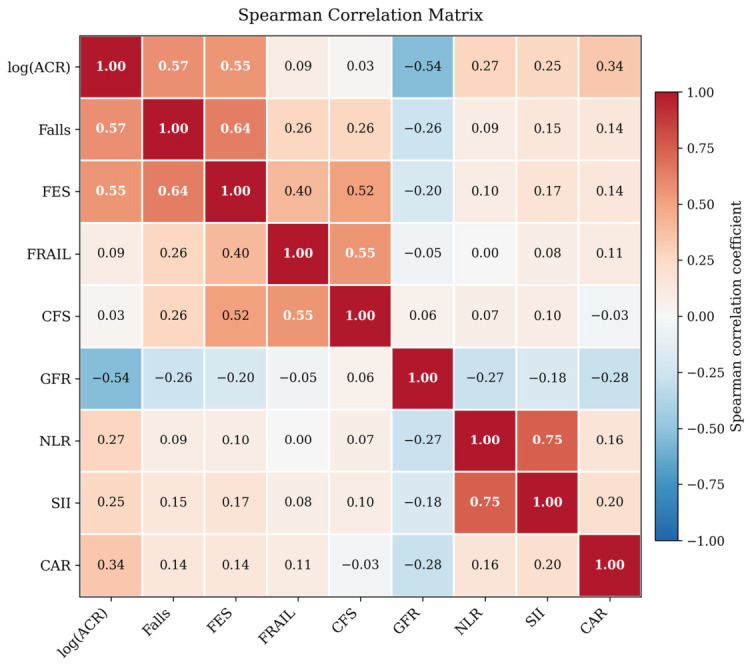
Spearman correlation matrix heatmap of ACR, geriatric outcomes, inflammatory indices, and laboratory parameters. *n* = 295.

Log-ACR correlated strongly and positively with fall count (rho = 0.575, *p* < 0.001) and FES score (rho = 0.552, *p* < 0.001; Figure 4 and Figure 5). Correlations with frailty scores were weak and non-significant (FRAIL: rho = 0.090, *p* = 0.127; CFS: rho = 0.031, *p* = 0.601). Log-ACR correlated significantly with all inflammatory indices: NLR (rho = 0.274), SII (rho = 0.257), and CAR (rho = 0.342; all *p* < 0.001; Figure 6).

### 3.4. Hierarchical Logistic Regression: Falls

In hierarchical logistic regression, log-ACR was consistently and strongly associated with falls across all adjustment levels (Table 1). In the crude model, each unit increase in log-ACR was associated with a 2.77-fold increase in fall odds (OR 2.77, 95% CI 2.14–3.83). After adjustment for age and sex, the association strengthened (OR 2.97, 95% CI 2.25–4.31) and held firm in the fully adjusted model (OR 3.84, 95% CI 2.74–6.65). VIF analysis confirmed no multicollinearity for the primary exposure (logACR, VIF = 2.98); elevated values for age (VIF = 16.89) and GFR (VIF = 7.31) reflected the expected age–GFR correlation in geriatric populations (Table S2). When ACR was entered categorically with A1 as reference, A2 showed an OR of 9.51 (95% CI 5.21–25.85), and A3 showed an OR of 88.96 (95% CI 27.74–very large). For A3, the extremely high OR reflects near-complete separation in the data (93.2% fall rate) and should be interpreted with caution.

### 3.5. Logistic Regression: Frailty

While bivariate associations between ACR and frailty were non-significant, fully adjusted models uncovered consistent independent associations across both frailty definitions and cut-offs (Table 2). For FRAIL ≥ 3 (frail), log-ACR achieved significance only in the fully adjusted model (OR 1.41, 95% CI 1.05–2.03, *p* = 0.028). The association also reached significance at the pre-frail threshold (FRAIL ≥ 2: fully adjusted OR 1.49, 95% CI 1.08–2.21). The CFS followed the same pattern: frail (CFS ≥ 5) showed a fully adjusted OR of 1.87 (95% CI 1.38–2.83, *p* = 0.002), and pre-frail (CFS ≥ 4) showed a fully adjusted OR of 1.37 (95% CI 1.02–1.93). This pattern, significance emerging only after full adjustment, was consistent across all four frailty outcomes, suggesting that confounders, particularly age and GFR, were masking the independent contribution of albuminuria to frailty.

### 3.6. Subgroup and Sensitivity Analyses

**Figure 7:**
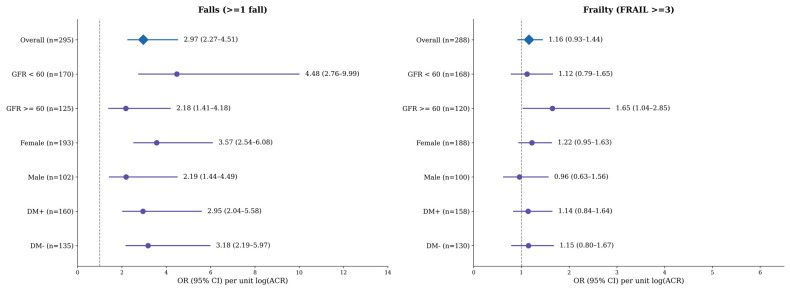
Forest plot showing subgroup associations between log(ACR) and falls (**left panel**) and frailty (FRAIL ≥ 3, (**right panel**)). OR (95% CI) adjusted for age and sex. The reference line is at OR = 1. The ACR–fall association was consistent and significant across all subgroups, whereas the ACR–frailty association was weaker and reached significance only in the GFR ≥ 60 subgroup. The blue diamond represents the overall estimate, and the purple circles represent subgroup-specific estimates. Horizontal lines indicate 95% confidence intervals; the vertical dashed line denotes the null value (OR = 1).

The ACR–fall association was consistent across all subgroups (Figure 7, left panel). The effect was stronger in GFR < 60 (OR 4.48, 95% CI 2.76–9.99) compared to GFR ≥ 60 (OR 2.18, 95% CI 1.41–4.18), in women (OR 3.57) compared to men (OR 2.19), and similar regardless of DM status. For frailty, the subgroup pattern differed (Figure 7, right panel): the ACR–frailty association was weaker and non-significant in most subgroups (overall age–sex adjusted OR 1.16, 95% CI 0.93–1.44), but notably reached significance in the GFR ≥ 60 subgroup (OR 1.65, 95% CI 1.04–2.85), suggesting that albuminuria may carry additional prognostic value for frailty, specifically in patients with preserved kidney function. In the sensitivity analysis restricted to GFR < 60 (*n* = 170), the dose–response for falls was even more pronounced: A1 29.6%, A2 69.0%, A3 95.1%.

### 3.7. ROC Analysis

**Figure 8:**
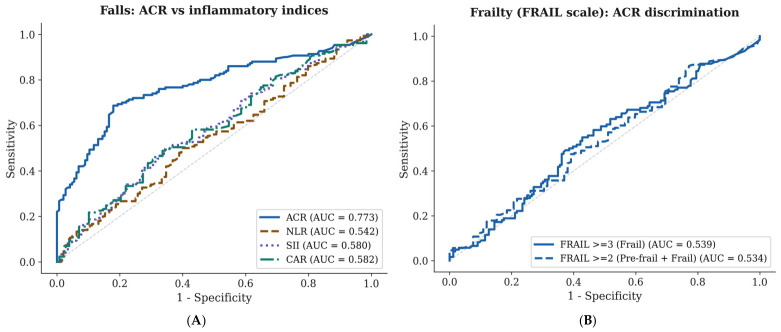

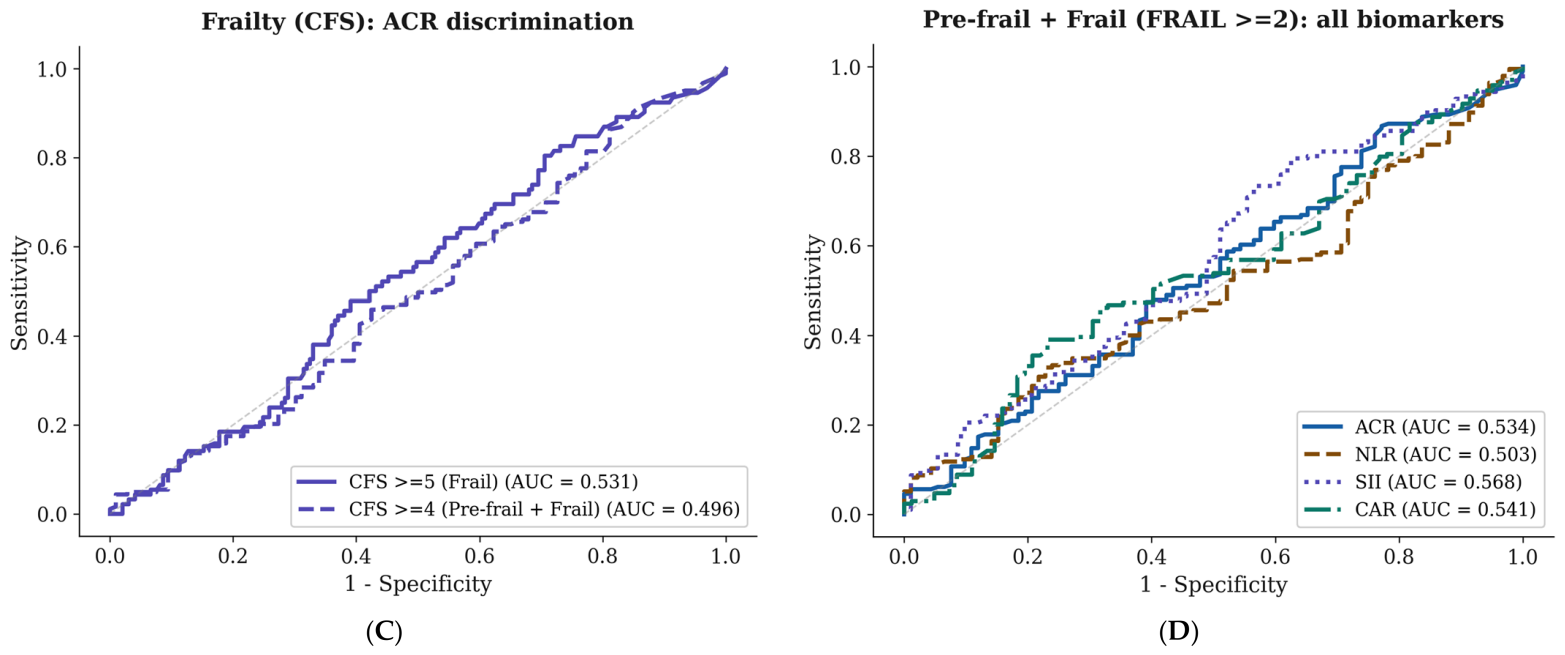
(**A**). ROC curves for fall prediction. ACR (AUC 0.773) clearly outperformed NLR (0.542), SII (0.580), and CAR (0.582). The optimal ACR cut-off was 21.70 mg/g (sensitivity 0.687, specificity 0.821). (**B**). ROC curves for FRAIL scale frailty. ACR discrimination was poor at both thresholds: FRAIL ≥ 3 (AUC 0.539) and FRAIL ≥ 2 (AUC 0.534). (**C**). ROC curves for CFS frailty. ACR discrimination: CFS ≥ 5 (AUC 0.531) and CFS ≥ 4 (AUC 0.496). (**D**). ROC curves for pre-frail + frail (FRAIL ≥ 2) prediction using all biomarkers. None exceeded AUC 0.57.

We evaluated the discriminative ability of ACR and inflammatory indices across multiple outcome definitions using ROC analysis (Figure 8, Table 3).

For falls, ACR showed good discriminative ability (AUC 0.773, 95% CI 0.718–0.825), clearly outperforming NLR (0.542), SII (0.580), and CAR (0.582) (Figure 8A). At the optimal Youden-index cut-off of 21.70 mg/g, sensitivity was 0.687 and specificity 0.821. This cut-off sits below the conventional KDIGO A2 threshold of 30 mg/g, suggesting that geriatric risk may begin at lower albuminuria levels than those defining kidney damage progression.

For frailty, ACR discrimination was poor regardless of instrument or threshold. Using the FRAIL scale, AUC was 0.539 for frail (≥3) and 0.534 for pre-frail + frail (≥2) (Figure 8B). Using the CFS, AUC was 0.531 for frail (≥5) and 0.496 for pre-frail + frail (≥4) (Figure 8C). These values barely exceeded the chance line, meaning that ACR alone cannot serve as a frailty screening tool despite its significance in multivariable models.

Inflammatory indices performed no better for frailty prediction: FRAIL ≥ 2, NLR (0.503), SII (0.568), and CAR (0.541) all remained near the diagonal (Figure 8D). The gap between ACR’s strong regression-based association with falls and its weak ROC performance for frailty points to a key distinction: ACR contributes meaningful information within a multivariate risk model for frailty but lacks standalone predictive power when used in isolation.

The clear separation between ACR and the three inflammatory indices carries a practical message: while NLR, SII, and CAR reflect systemic inflammation that correlates with albuminuria, they add little discriminative value for fall prediction beyond what ACR already provides. A single spot urine ACR measurement outperformed a panel of blood-based inflammatory biomarkers for this purpose.

Turning to frailty, ACR discrimination was poor regardless of the FRAIL scale threshold used. For the frail definition (FRAIL ≥ 3), AUC was only 0.539, and lowering the threshold to include pre-frail patients (FRAIL ≥ 2) did not improve performance (AUC 0.534). Both curves hugged the diagonal, confirming that ACR alone cannot identify frail or pre-frail patients by this instrument.

This near-diagonal performance raises an important question: If ACR independently predicts frailty in multivariable regression (fully adjusted OR 1.41, *p* = 0.028), why does it fail as a standalone discriminator? The answer lies in the distinction between statistical association and predictive discrimination. ACR captures a small but real component of frailty risk that becomes visible only after removing the variance explained by age, GFR, and comorbidities. In isolation, however, this signal is too weak to separate frail from non-frail patients.

The CFS yielded the same pattern. ACR discrimination for CFS ≥ 5 (frail) was 0.531 and for CFS ≥ 4 (pre-frail + frail) dropped further to 0.496, essentially at the level of chance. The concordance between two independent frailty instruments strengthens the conclusion that ACR’s contribution to frailty prediction operates through multivariate pathways rather than as a standalone marker.

The convergence of Figure 8B,C provides validation: two independently administered frailty instruments, one self-reported (FRAIL) and one clinician-rated (CFS), both confirm that ACR lacks standalone screening utility for frailty. This consistency across instruments rules out the possibility that the poor ROC performance reflects limitations of a single frailty tool.

Finally, we compared all four biomarkers for their ability to identify pre-frail and frail patients (FRAIL ≥ 2). None exceeded an AUC of 0.57: ACR (0.534), SII (0.568), CAR (0.541), and NLR (0.503). This uniformly weak performance suggests that frailty in this cohort is driven by factors, such as sarcopenia, cognitive decline, and social determinants, that simple blood or urine biomarkers do not capture well in isolation.

Taken together, these four panels reveal a striking contrast in ACR’s clinical utility: it is a strong, clinically actionable biomarker for fall risk (AUC 0.773) but not for frailty (AUC 0.50–0.54). This dissociation suggests that albuminuria connects to falls through relatively direct pathways, possibly involving microvascular dysfunction, orthostatic hemodynamics, and neuromuscular impairment, whereas frailty arises from a broader constellation of biological, psychological, and social factors that no single biomarker can adequately capture.

## 4. Discussion

In this exploratory cross-sectional study of 295 older adults attending nephrology and geriatrics clinics, we evaluated whether albuminuria levels are associated with falls, fear of falling, and frailty in predialysis CKD patients. Three principal findings emerged. First, fall prevalence showed a clear dose–response gradient across KDIGO albuminuria categories (A1: 31.2%, A2: 72.0%, A3: 93.2%), and this association remained strong after full multivariable adjustment (OR 3.84 per log-unit ACR increase). Second, albuminuria independently predicted frailty by both the FRAIL scale and CFS, but only after adjustment for confounders, a suppression effect suggesting that age and GFR mask the independent contribution of albuminuria. Third, despite its strong regression-based associations, ACR showed good discriminative ability only for falls (AUC 0.773) and not for frailty (AUC 0.50–0.54), establishing that its clinical utility as a screening tool is limited to fall risk.

These findings extend the existing literature by simultaneously examining falls, fear of falling, and frailty using dual-validated instruments in a predialysis CKD cohort, a combination not previously reported from Turkey or the broader Middle Eastern region.

### 4.1. Albuminuria and Falls

Our finding of a fully adjusted OR of 3.84 per log-unit ACR increase for falls extends the seminal work of Bowling et al., who reported that albuminuria (ACR ≥ 30 mg/g) was associated with serious fall injuries (OR 1.39, 95% CI 1.10–1.75) in the REGARDS cohort [6]. The substantially larger effect size in our study likely reflects our focus on older, sicker patients (median age 73, 57.6% GFR < 60) compared to the REGARDS population (mean age 69, community-dwelling). Britting et al., in the SCOPE study of 2256 European adults aged 75 and older, linked impaired kidney function to falls but focused on eGFR rather than albuminuria [29]. Sedaghat et al., studying 4081 Rotterdam Study participants, found that lower eGFR was associated with altered gait but not directly with fall incidence after adjustment, suggesting that albuminuria may be the more clinically relevant biomarker [30]. These findings underscore the need for closer collaboration between nephrology and geriatric services to address the overlapping risks of kidney disease and functional decline [31].

Our ROC-derived optimal cut-off of 21.70 mg/g sits below the conventional KDIGO A2 threshold of 30 mg/g. This finding aligns with Chang et al., who demonstrated that even low-grade albuminuria (ACR < 30 mg/g) was associated with pre-frailty/frailty (OR 1.13, 95% CI 1.01–1.27) in the I-Lan Longitudinal Aging Study [20]. We propose that for geriatric risk assessment, the current KDIGO thresholds may be insufficiently sensitive, and a lower ACR threshold warrants investigation in prospective studies.

### 4.2. Albuminuria and Frailty

We observed a suppression effect: ACR–frailty links surfaced only after full adjustment, mirroring Bongetti et al. in the ASPREE trial, the largest dedicated analysis to date [21]. Among 14,195 initially healthy older adults, higher ACR tertiles were significantly associated with incident frailty (HR 1.34, 95% CI 1.09–1.65), while eGFR was not independently predictive. Our study extends this by using dual frailty assessment tools: the self-reported FRAIL scale and the clinician-rated CFS, which yielded concordant results (fully adjusted OR 1.41 and 1.87, respectively). This convergent validity strengthens confidence in the finding.

Mielke et al., in the Berlin Initiative Study (1076 very old adults, mean age 82), showed that albuminuria independently predicted frailty worsening and death, with effect sizes increasing across KDIGO categories [19]. Huang et al., in a 2025 prospective Taiwanese CKD cohort, found that frailty defined by the 80-item Frailty Index predicted adverse composite outcomes in moderate-to-severe CKD [32]. Chang et al. similarly identified proteinuria as an independent predictor of frailty among adults with metabolic syndrome in the NHANES III cohort [33]. Our study contributes the first evidence from a Turkish population using FRAIL and CFS simultaneously, demonstrating that the albuminuria–frailty association is generalizable across different frailty operationalizations and ethnic contexts.

Falls and frailty are not isolated events but indicators of broader vulnerability; Gomez–Ramos et al. (2024) demonstrated that coexistence of multiple geriatric syndromes was independently associated with in-hospital mortality [34]. Heybeli et al. identified polypharmacy, diabetes, and low hemoglobin as key fall risk factors in elderly CKD patients, consistent with our finding of clustering these factors in the A3 category [35]. Gait abnormalities represent a mechanistic link; Bowling et al. showed that CKD patients exhibit slower gait speed, shorter stride length, and greater gait variability [36].

### 4.3. Role of Inflammatory Indices

ACR correlated significantly with all three inflammatory indices (NLR: rho 0.274, SII: 0.257, CAR: 0.342), supporting the hypothesis that systemic inflammation mediates part of the albuminuria–geriatric outcome relationship. However, the inflammatory indices themselves showed poor discriminative ability for falls (AUC 0.54–0.58), implying that inflammation represents a shared pathway, but ACR captures additional non-inflammatory mechanisms including microvascular dysfunction, endothelial injury, and sarcopenic pathways. Tang et al. reported from 16,705 NHANES participants that all CBC-derived inflammatory markers correlated positively with frailty, with NLR showing a mortality HR of 1.73 [15]. Zeng et al. identified NLR and CRP as independent frailty risk factors with combined AUC exceeding 0.84 in hospitalized elderly [37]. Our lower values likely reflect our outpatient setting and the relatively lower inflammatory burden of predialysis patients compared to hospitalized cohorts.

ACR captures not only inflammatory pathways but also non-inflammatory mechanisms including microvascular dysfunction, endothelial glycocalyx degradation, and orthostatic hemodynamic changes that CBC-derived indices miss. These indices reflect acute systemic inflammation rather than the chronic, tissue-level inflammatory milieu driving geriatric vulnerability [38].

CRP is a pleiotropic acute-phase reactant influenced by obesity, psychosocial stress, intercurrent infections, and medications. Gomez-Ramos et al. (2026) demonstrated significant associations between inflammatory biomarkers and psychological burden, illustrating how non-disease factors confound CRP-based indices [39]. Our uncontrolled heterogeneity may partly explain CAR’s poor performance.

### 4.4. Clinical Implications

These results have practical implications. ACR measurement, already routine in CKD monitoring, could serve a dual purpose: tracking nephropathy progression and screening for geriatric risk. We propose that patients with ACR ≥ 22 mg/g (our optimal cut-off, approximating the A2 threshold) should be referred for geriatric assessment including fall risk evaluation, exercise prescription, medication review for fall-inducing drugs, and environmental hazard assessment. This integration of nephrology and geriatric care aligns with the emerging nephrogeriatrics paradigm advocated by recent international guidelines [40].

### 4.5. Limitations

First, the cross-sectional design precludes causal inference. Longitudinal studies are needed to determine whether albuminuria reduction through nephroprotective interventions translates into fewer falls and attenuated frailty progression.

Second, ACR data were unavailable for 105 participants (26.3%); excluded participants had significantly higher GFR (median 80 vs. 55 mL/min, *p* < 0.001) and lower fall prevalence (32.3% vs. 50.8%, *p* = 0.002), confirming non-random missingness concentrated among lower-risk patients (Table S1). In routine clinical practice, spot urine samples may not be obtained from elderly patients due to compliance difficulties, urinary incontinence, or because ACR testing was not requested for patients with preserved kidney function. Compared with those lacking ACR data, our analytic cohort had lower GFR and higher fall rates, consistent with enrichment for higher-risk patients (Table S1).

Third, the single-center design from Kayseri, Turkey, with a predominantly female (65.4%) and low-education (45.4% illiterate) population may limit generalizability. This mirrors local demographics rather than selection bias. Cultural attitudes toward fall reporting, health literacy, and healthcare-seeking behavior may differ across populations, potentially affecting both exposure ascertainment (ACR measurement patterns) and outcome reporting (fall disclosure). Multi-center, multi-ethnic replication is needed.

Fourth, fall recall over 12 months is subject to recall bias. Prospective fall diaries or electronic monitoring would provide more accurate data. Studies suggest that retrospective self-report underestimates true fall rates by approximately 13–25% in older adults compared with prospective diaries.

Fifth, the fully adjusted model showed multicollinearity between age and GFR (VIF 16.89 and 7.31, respectively), an inherent feature of geriatric CKD populations where kidney function declines with age. Comorbidity count also showed structural overlap with individual comorbidity variables (VIF = 13.65). Because both age and GFR are clinically indispensable confounders, all covariates were retained; however, their individual coefficient estimates should be interpreted with caution. The primary exposure, logACR (VIF = 2.98), was unaffected (Table S2).

Sixth, the inclusion of patients with GFR ≥ 60 (42.4%) expanded the population beyond strict predialysis CKD. However, the sensitivity analysis restricted to GFR < 60 yielded even stronger associations, reinforcing the findings in the target population.

Seventh, the single spot urine ACR measurement introduces day-to-day variability. KDIGO guidelines recommend confirmation with repeat testing, which was not systematically performed. Although nephrology clinic patients had clinically documented CKD, and reduced eGFR among geriatrics clinic participants was verified from prior clinical records, repeat ACR confirmation was not systematically available for geriatrics clinic participants with preserved eGFR. Therefore, albuminuria-based CKD classification in the preserved-eGFR subgroup, which comprised 42.4% of the analytic cohort, should be interpreted cautiously.

Eighth, the extremely high OR for A3 versus A1 (88.96) reflects near-complete separation (93.2% fall rate in A3) and should be interpreted as indicating a very strong association rather than a precise point estimate.

Ninth, recruitment was conducted over a three-month period (April–June 2025), which may limit the representativeness of the sampled outpatient population. However, fall history was ascertained for the preceding 12 months and therefore included all seasons; thus, the main concern for fall ascertainment is recall bias rather than direct seasonal under-ascertainment.

Tenth, the optimal ACR cut-off of 21.70 mg/g was derived from internal ROC analysis without external validation. This cut-off is susceptible to optimism bias and requires validation in independent cohorts before clinical implementation.

### 4.6. Strengths

This is the first study from Turkey to evaluate the albuminuria–falls–frailty relationship in CKD patients using dual-validated geriatric assessment tools. The hierarchical regression approach with three adjustment levels provides transparent assessment of confounding. The inclusion of novel inflammatory indices extends the analysis beyond traditional risk factors. The head-to-head comparison of ACR discrimination for falls versus frailty addresses clinically relevant questions.

## 5. Conclusions

Albuminuria is a strong, independent, and graded predictor of falls in older patients with CKD, with each 10-fold increase in ACR associated with a nearly 4-fold increase in fall odds after multivariable adjustment. The dose–response relationship across KDIGO categories (A1: 31%, A2: 72%, A3: 93%) is among the strongest reported for any single biomarker in the fall risk literature. While ACR also independently predicts frailty after multivariable adjustment, its discriminative ability for frailty screening is limited. We recommend integrating ACR-based geriatric risk stratification into routine nephrology practice, with an ACR threshold of approximately 22 mg/g triggering comprehensive fall risk assessment. Prospective studies and intervention trials are warranted to determine whether albuminuria-guided geriatric screening can reduce fall burden in CKD populations. These hypothesis-generating findings warrant confirmation in prospective, multi-center cohort studies. ACR-based screening in nephrology practice should focus specifically on fall risk rather than frailty, for which multidimensional geriatric assessment remains indispensable.

## Figures and Tables

**Table 1:** Hierarchical Logistic Regression: ACR as Predictor of Falls (*n* = 295).

Predictor	Model 0 (Crude)	Model 1 (Age + Sex)	Model 2 (Full)	*p* (Model 2)
**log(ACR), per unit**	2.77 (2.14–3.83)	2.97 (2.25–4.31)	3.84 (2.74–6.65)	<0.001
**A2 vs. A1 (ref)**	−	−	9.51 (5.21–25.85)	<0.001
**A3 vs. A1 (ref)**	−	−	88.96 (27.74–large)	<0.001

OR (95% CI) from bootstrap (1000 resamples). Model 2 adjusted for age, sex, GFR, DM, HT, polypharmacy, comorbidity count, and education.

**Table 2:** Hierarchical Logistic Regression: ACR as Predictor of Frailty.

Outcome	Model 0 (Crude)	Model 1 (Age + Sex)	Model 2 (Full)	*p* (Model 2)
**FRAIL** ≥ **3 (*n* = 288)**				
log(ACR)	1.11 (0.89–1.37)	1.16 (0.93–1.45)	1.41 (1.05–2.03)	0.028
**CFS** ≥ **5 (*n* = 289)**				
log(ACR)	1.08 (0.86–1.33)	−	1.87 (1.38–2.83)	0.002

Model 2 adjusted for age, sex, GFR, DM, HT, polypharmacy, comorbidity count, and education. OR (95% CI) from bootstrap.

**Table 3:** ROC Analysis: Discriminative Performance for Falls and Frailty.

Predictor	AUC (95% CI)	Cut-Off	Sens	Spec	PPV	NPV
**ACR** → **Falls**	0.773 (0.718–0.825)	21.70 mg/g	0.687	0.821	0.798	0.719
**NLR** → **Falls**	0.542 (0.482–0.604)	2.29	0.500	0.604	0.523	0.581
**SII** → **Falls**	0.580 (0.518–0.641)	578.85	0.493	0.660	0.565	0.585
**ACR** → **FRAIL** ≥ **3**	0.539 (0.470–0.606)	16.30 mg/g	0.549	0.572	0.486	0.638

## Data Availability

The datasets generated and analyzed during the current study are available from the corresponding author on reasonable request.

## Supplementary Materials

The following supporting information can be downloaded at: https://www.mdpi.com/article/10.3390/jcm15124772/s1, Table S1. Baseline Characteristics of the Study Population by KDIGO Albuminuria Category (*n* = 295). Comparison of baseline characteristics between included (*n* = 295) and excluded (*n* = 105) participants. Table S2. Variance Inflation Factor (VIF) Analysis for Covariates in the Fully Adjusted Logistic Regression Model (Model 2).
