# Supplementary material for: Albuminuria Levels and Geriatric Outcomes in Predialysis: Chronic Kidney Disease: Falls, Fear of Falling, and Frailty in a Cross-Sectional Study
Source: J Clin Med. 2026 Jun 19;15(12):4772. doi: 10.3390/jcm15124772 (PMC13302223; doi:10.3390/jcm15124772)
Supplement: Supplementary file 1 [file jcm-15-04772-s001.zip › jcm-4343240-supplementary.pdf]

Table S1. Baseline Characteristics of the Study Population by KDIGO Albuminuria Category (n = 295). Comparison of baseline characteristics between included (n = 295) and excluded (n = 105) participants.

Variable	Included (n = 295)	Excluded (n = 105)	p value
Demographics			
**Age, years**	73.0 (68.0-80.0)	74.0 (69.0-80.2)	0.341
**Female, n (%)**	193 (65.4)	77 (73.3)	0.172
**Education**			
Illiterate, n (%)	134 (45.4)	67 (63.8)	
Primary school, n (%)	101 (34.2)	6 (5.7)	
University, n (%)	33 (11.2)	21 (20.0)	
Clinical characteristics			
**GFR, mL/min/1.73 m2**	55.0 (39.0-77.0)	80.0 (60.0-89.0)	<0.001
GFR <60, n (%)	170 (57.6)	24 (22.9)	
GFR >=60, n (%)	125 (42.4)	79 (75.2)	
**DM, n (%)**	160 (54.2)	42 (40.0)	0.017
**HT, n (%)**	241 (81.7)	75 (71.4)	0.038
**Polypharmacy, n (%)**	151 (51.2)	62 (59.0)	0.203
Laboratory parameters			
**Creatinine, mg/dL**	1.1 (0.8-1.5)	0.8 (0.7-1.0)	<0.001
**Albumin, g/L**	43.0 (41.0-45.0)	44.0 (43.0-45.0)	<0.001
**CRP, mg/L**	3.2 (1.5-6.6)	2.5 (1.2-5.5)	0.063
**Hemoglobin, g/dL**	13.3 (12.1-14.3)	13.8 (12.9-14.6)	0.001
**NLR**	2.2 (1.7-2.8)	2.3 (1.7-3.0)	0.944
**SII**	527.7 (384.4-738.9)	534.0 (403.7-728.9)	0.753
Geriatric outcomes			
**Fallers (>=1 fall), n (%)**	150 (50.8)	32 (32.3)	0.002
**Fall count**	1.0 (0.0-2.0)	0.0 (0.0-1.0)	<0.001
**FES score**	40.0 (22.0-67.0)	39.0 (20.0-48.0)	0.130
**FRAIL score**	2.0 (1.0-3.0)	2.0 (0.0-3.0)	0.012
**CFS score**	4.0 (3.0-5.0)	4.0 (3.5-5.0)	0.063

Values are median (interquartile range) or n (%). P-values from Mann-Whitney U test (continuous) or chi-square test (categorical). Bold p-values indicate statistical significance ($p < 0.05$). CFS, Clinical Frailty Scale; CRP, C-reactive protein; DM, diabetes mellitus; FES, Falls Efficacy Scale; GFR, glomerular filtration rate; HT,

hypertension; NLR, neutrophil-to-lymphocyte ratio; SII, systemic immune-inflammation index.

Table S2. Variance Inflation Factor (VIF) Analysis for Covariates in the Fully Adjusted Logistic Regression Model (Model 2)

Covariate	VIF	Tolerance (1/VIF)	Interpretation
**log(ACR)**	2.98	0.336	Acceptable
**Age, years**	16.89	0.059	High *
**Sex (female)**	3.12	0.321	Acceptable
**GFR, mL/min/1.73 m2**	7.31	0.137	Elevated *
**DM**	3.56	0.281	Acceptable
**HT**	9.10	0.110	Elevated *
**Polypharmacy (>=4 medications)**	3.05	0.328	Acceptable
**Comorbidity count**	13.65	0.073	High *
**Education (ordinal)**	1.67	0.599	Acceptable

** VIF > 5 indicates potential multicollinearity. VIF > 10 indicates high multicollinearity. ACR, albumin-to-creatinine ratio; DM, diabetes mellitus; GFR, glomerular filtration rate; HT, hypertension; VIF, variance inflation factor. As a sensitivity check, removing comorbidity count from the model (retaining individual comorbidities DM and HT) reduced VIF for HT to 6.04 but age (16.63) and GFR (7.29) remained elevated, confirming that the age-GFR correlation is an inherent feature of geriatric CKD populations rather than a model specification error.*
